# Influence of Treg cells and HBV genotype on sustained response and drug resistance in the treatment with nucleoside drugs

**DOI:** 10.1590/1414-431X20165796

**Published:** 2017-03-02

**Authors:** Y.R. Zhang, B. Li, C.X. Wang, N. Zhou, W. Qi, X.L. Li, L.Y. Wu, S.F. Wei, Y.D. Zhang

**Affiliations:** 1 Department of Infectious Diseases, The First People’s Hospital of Lanzhou, Gansu, China; 2 Department of Thoracic Surgery, Gansu Province Tumor Hospital, Gansu, China; 3 Department of Geriatrics, The First People’s Hospital of Lanzhou, Gansu, China

**Keywords:** Hepatitis B chronic, CD4^+^CD25^+^, CD8^+^CD28^-^, Regulatory T cells, Viral response, Nucleoside

## Abstract

We aimed to investigate the influence of regulatory T cells including CD4^+^CD25^+^, CD8^+^CD28^-^ and hepatitis B virus (HBV) genotype on sustained virological response and tolerance of nucleoside drugs. One hundred and thirty-seven patients were enrolled. Lamivudine was administered to 84 patients. Entecavir was administered to the other 53 patients. Before treatment, biochemical tests, HBV DNA load, HBV serum level, HBV genotype, PB CD3^+^, CD4^+^, CD8^+^, CD4^+^CD25^+^/CD3^+^, and CD8^+^CD28^-^/CD3^+^ frequencies were measured. Based on HBV DNA loads after 4 weeks of therapy, patients were divided into response group and suboptimal response group. The lamivudine group received treatment continuously, and then patients were categorized into non-resistance group and resistance group. Compared with the suboptimal response and resistance groups for lamivudine, CD4^+^CD25^+^/CD3^+^ levels were higher in the response and non-resistance groups (t=4.372, P=0.046; t=7.262, P=0.017). In the non-resistance group, CD8^+^CD28^-^/CD3^+^ frequency was lower than in the resistance group (t=5.527, P=0.037). Virus load and hepatitis B E antigen (HBeAg)-positive rate were significantly lower than in the response and resistance group (t=2.164, P=0.038; *X* ^2^=4.239, P=0.040; t=2.015, P=0.044; *X* ^2^=16.2, P=0.000). Incidence of drug resistance was high in patients with virogene type C. For the virological response to entecavir, CD8^+^CD28^-^/CD3^+^ level was significantly lower than that of the suboptimal response group (t=6.283, P=0.036). Response and suboptimal response groups were compared in CD3^+^, CD4^+^, CD8^+^, CD4^+^CD25^+^/CD3^+^ and virus genotype, and differences were not statistically significant (P>0.05). Baseline regulatory T cells including CD4^+^CD25^+^/CD3^+^ and CD8^+^CD28^-^/CD3^+^ frequencies have a relationship with the incidence of rapid virological response and the resistance to nucleoside drugs. Patients with HBV genotype C receiving lamivudine more often underwent drug resistance. Antiviral efficacy and the resistance to lamivudine were closely correlated with baseline factors; the same cannot be found for entecavir.

## Introduction

It is known that antiviral efficacy and drug resistance are related to chronic hepatitis B virus (HBV). The host’s immune state, the selection of nucleoside drugs and the virus’ genotype could be major factors in antiviral efficacy and drug resistance in patients (1,2). Recent studies have indicated that regulatory T cells (Tregs) inhibited effector T cells in the HBV infection process, and consequently the virus was not completely eliminated (3). CD4^+^CD25^+^ and CD8^+^CD28^-^ are Treg subgroups that have mostly been studied recently. Stoop et al. (4) found that PB CD4^+^CD25^+^ Treg proportion in patients with chronic HBV was obviously increased compared to healthy control patients and healed patients, and the proportion of patients with positive hepatitis B E antigen (HBeAg) was higher than patients with negative HBeAg. Franzese et al. (5) evaluated Tregs levels among patients that carried asymptomatic viruses, patients at the chronic infection stage, patients with previous infections, and healthy controls, and found no significant difference. This was independent of HBeAg status, HBV load and different antiviral therapies. However, Zhang et al. (6) reported that the proportion of PB CD4^+^CD25^+^ was significantly reduced after HBV DNA was inhibited by entecavir, and the immune function of patients was recovered. In addition, other studies found that the high expression of CD8^+^CD28^-^ was a marker for decreased T cell function, which may go against the clearance of HBV (7,8). However, there are no studies that determined whether or not CD4^+^CD25^+^ and CD8^+^CD28^-^ levels have an influence on antiviral efficacy and drug resistance to nucleoside drugs.

The HBV genotype is divided into nine types from A to I. In general, patients with HBV infection have mostly types A and B, while Chinese patients with HBV infection have mostly types B and C (9). Studies have reported that the virological response to lamivudine therapy was more likely to occur in patients with genotype B than in patients with genotype C, which present with more severe conditions and poor drug efficacy (10). To date, no studies exist with respect to the relationship between virus genotypes and early virological responses to nucleoside drug treatment (lamivudine and entecavir).

Therefore, this study aimed to investigate the influence of Treg cells at baseline and virus genotype on early virological response and drug resistance to nucleoside drugs.

## Patients and Methods

### Patients

This study included a total of 137 inpatients and outpatients with chronic HBV infection from May 2010 to April 2014. Among these patients, 95 were males and 42 were females with age ranging between 24-70 years (mean age: 46.8 years). The Guidelines for the Prevention and Treatment of Chronic HBV Infection, established by the Chinese Society of Hepatology and the Society of Infectious Diseases, was used as the diagnostic criteria (11). Based on these guidelines, study participants were classified as 88 patients with chronic HBV, 49 patients with cirrhosis, 54 HBeAg-positive patients, and 83 HBeAg-negative patients (demographic data are reported in Table 1). Inclusion criteria were as follows: 1) patients with positive HBsAg for more than 6 months, and HBV DNA ≥10^4^ cps/mL in two examinations (reference value: <1×10^3^ cps/mL); 2) patients with alanine aminotrasferase (ALT) levels ≥1.5 ULN; 3) patients treatment-naive for lamivudine and entecavir.

**Table:**
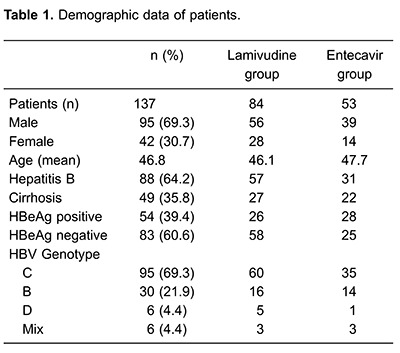

Exclusion criteria were as follows: patients with hepatitis C, hepatitis D, HIV infection, primary liver cancer and hepatic failure.

### Methods

Among the 137 patients with chronic HBV infection, 84 were administered 100 mg/day of lamivudine *po* (GlaxoSmithKline Medical, China), and 53 patients were administered 0.5 mg/day of entecavir (Chiatai Tianqing Pharmaceutical, China) *po* for treatment, according to the time sequence of hospitalization. Biochemical tests, HBV DNA burden, HBV serum level, HBV genotype, CD3^+^, CD4^+^, CD8^+^, CD4^+^CD25^+^/CD3^+^ and CD8^+^CD28^-^/CD3^+^ percentages were measured before treatment; biochemical tests and HBV DNA load were rechecked at the 4th, 12th and 24th week of treatment. During lamivudine therapy, if HBV DNA load rebounded, drug resistance was determined. Biochemical examination was performed using an automatic biochemical analyzer in The First People's Hospital of Lanzhou City, and the reagent for HBV DNA load was provided by Hunan Sansure Biotech Reagent Co., Ltd., China, with a lower limit of 500 IU/mL. HBV genotype and the test for drug resistance to lamivudine were performed using real-time PCR, and assisted by Xi'an KingMed Diagnostics, China. CD3^+^, CD4^+^, CD8^+^, CD4^+^CD25^+^/CD3^+^ and CD8^+^CD28^-^/CD3^+^ were detected using flow cytometry, and assisted by Xi'an KingMed Diagnostics.

Flow cytometry detection method was as follows: Elbow venous blood was collected early in the morning on an empty stomach, and was kept in sodium citrate anticoagulation tubes. Empty tubes were coded, and 20 µL of CD25-PE, CD28-PC7, CD8-FITC, CD4-PC5 and CD3-ECD monoclonal antibodies were added. Then, 100 µL of whole blood with anticoagulant was added and gently mixed. Afterwards, the tubes were placed at room temperature for 15 min, and BD general hemolysin was added and left for 10 min until complete specimen hemolysis. Then, the solution was centrifuged at 500 *g* for 5 min at 18-22°C. The supernatant was removed, calf serum washing liquid was added for rinsing, and centrifuged at 500 *g* for 5 min at 18-22°C. The supernatant was removed and fixed liquid was added to re-suspend cells for detection using a computer. A flow cytometry instrument (Beckman Coulter, model: FC500 MCL, USA) was used for detection, the reagent was provided by Beckman Coulter, and the CXP analysis software (USA) was used for data analysis.

### Grouping criteria

Patients were divided into two groups: the response group and the suboptimal response group, based on whether or not HBV DNA load was detected at the end of the 4th week of lamivudine or entecavir treatment. The comparison was performed between groups in terms of CD3^+^, CD4^+^, CD8^+^, CD4^+^CD25^+^/CD3^+^ and CD8^+^CD28^-^/CD3^+^ levels at baseline and the constituent ratio of the virus genotype. Patients in the lamivudine treatment group received treatment continuously for 96 weeks. Patients of this group were categorized into two groups, the resistance group and the non-resistance group, based on whether the patient was resistant to the drug during treatment or not. The resistance group was compared to the non-resistance group in terms of CD3^+^, CD4^+^, CD8^+^, CD4^+^CD25^+^/CD3^+^ and CD8^+^CD28^-^/CD3^+^ levels, as well as the constituent ratio of virus genotype.

### Statistical analysis

SPSS 19.0 (IBM, USA) software package was used for data processing. Data are reported as means±SD. The comparison of means between groups was performed using the Student's *t*-test, and the comparison of the constituent ratio was carried out using the X^2^ test. P<0.05 was considered to be statistically significant.

## Results

### Correlation between CD3^+^, CD4^+^, CD8^+^, CD4^+^CD25^+^,CD8^+^CD28^-^ levels and virological response to lamivudine therapy at the 4th week

Table 2 shows that, in the response group, the CD4^+^CD25^+^ level was higher than the suboptimal response group, and the difference was statistically significant (t=4.372, P=0.046). However, the CD8^+^CD28^-^ level was lower than in the suboptimal response group, and the difference was not significant (t=2.290, P=0.151). The differences between groups for CD3^+^, CD4^+^ and CD8^+^ levels were not significant (P>0.05).

**Table:**
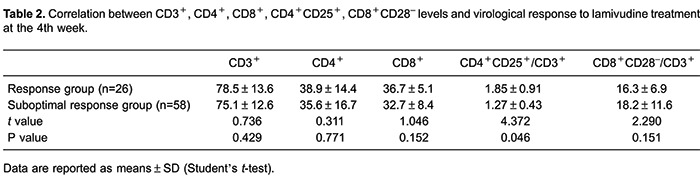

### Correlation between CD3^+^, CD4^+^, CD8^+^, CD4^+^CD25^+^, CD8^+^CD28^-^ levels and the incidence of drug resistance to lamivudine therapy at the 96th week

Table 3 shows that the levels of CD4^+^CD25^+^ and CD8^+^CD28^-^ were significantly different (t=7.262, P=0.017; t=5.527, P=0.037, respectively). The levels of CD3^+^, CD4^+^ and CD8^+^ were not significantly different (P>0.05).

**Table:**
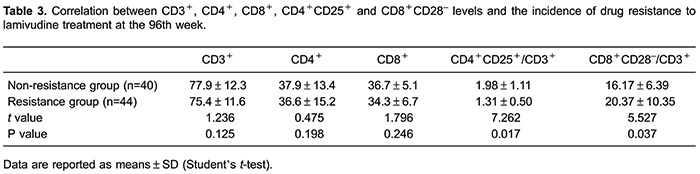

### Correlation between HBV genotype and virological response to lamivudine at the 4th week of treatment

As reported in Table 4, the proportions of HBV genotype C in the response and suboptimal response groups were 69.2 and 72.4%, respectively; the proportions of HBV genotype B in both groups were 19.2 and 20.0%, respectively; and the proportions of HBV genotype D in both groups were 7.7 and 5.2%, respectively. The constituent ratios of virus genotypes in both groups were compared, and the difference was not significant (X^2^=0.226, P=0.973). HBV DNA burden (to obtain the Lg value) in the response group was lower than that in the suboptimal response group, and the difference was significant (t=2.164, P=0.038). HBeAg-positive rate in the response group was reduced compared with the suboptimal response group, and the difference was significant (X^2^=4.239, P=0.040). The difference of ALT level in both groups was not significant.

**Table:**
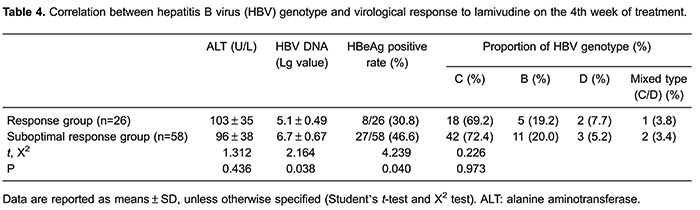

### Correlation between HBV genotype and the incidence of drug resistance at the 96th week of lamivudine treatment

As reported in Table 5, the proportions of HBV genotype C in non-resistance and resistance groups were 60 and 79.5%, respectively; 30 and 2.3% for genotype B, respectively; and 7.5 and 2.3% for genotype D, respectively. The constituent ratios of virus genotypes in both groups were compared, and the difference was statistically significant (X^2^=59.714, P=0.000). In the non-resistance group, HBV DNA burden (to obtain the Lg value) and HBeAg-positive rate were lower than in the resistance group, and the differences were statistically significant (t=2.015, P=0.044; X^2^=16.2, P=0.000, respectively).

**Table:**
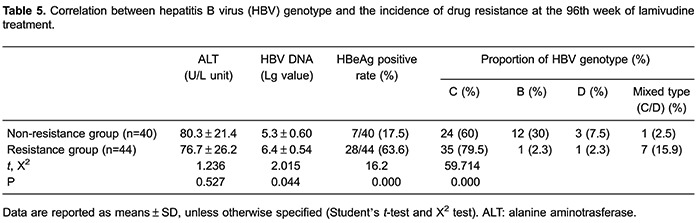

### Correlation between CD3^+^, CD4^+^, CD8^+^, CD4^+^CD25^+^, CD8^+^CD28^-^ levels and virological response to entecavir treatment at the 4th week

Table 6 indicates that CD8^+^CD28^-^/CD3^+^ level in the response group was lower than the suboptimal response group, and the difference was statistically significant (t=6.283, P=0.036). The differences in CD3^+^, CD4^+^, CD8^+^ and CD4^+^CD25^+^/CD3^+^ levels between both groups were not significant (P>0.05).

**Table:**
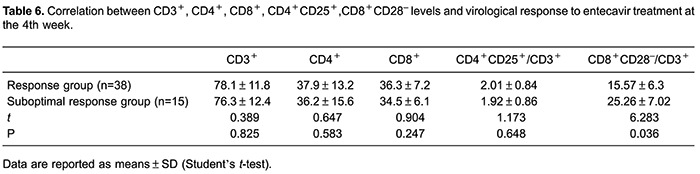

### Correlation between HBV genotype and virological response to entecavir at the 4th week of treatment

As reported in Table 7, the proportions of HBV in the response and suboptimal response groups were 65.8 and 66.7% for genotype C, respectively; 26.3 and 26.7.3% for genotype B, respectively, and 2.6 and 0% for genotype D, respectively. Comparisons were performed in terms of HBV DNA loads (to obtain the Lg value), HBeAg positive rate, ALT level and the constituent ratio of virus genotypes, and the differences were not significant (P>0.05).

**Table:**
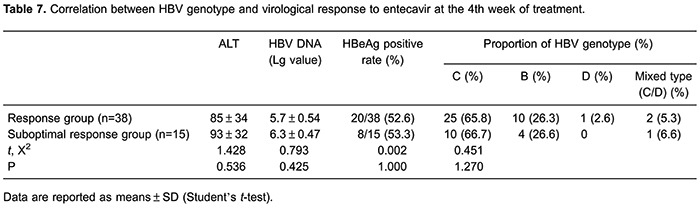

## Discussion

Recent international and local studies have indicated that early virological response could predict drug resistance to nucleoside analogues in the future (12). In the current study, HBeAg positive rate and HBV DNA load in the virological response group and non-resistance group were lower than in the suboptimal response group and drug resistance group at the 4th week of lamivudine therapy. This indicates that negative HBeAg and low viral replication are advantage factors for the four-week virological response to lamivudine therapy and non-drug resistance at the 96th week of treatment. Virological response is more likely to occur in patients with HBV infection and negative HBeAg, which may be related to immune statuses that are different from patients with positive HBeAg, in addition to low viral replication. The results of this study revealed that patients with virus genotype C were more prone to drug resistance following lamivudine therapy, which was similar to the results of studies performed by other Chinese scholars (13). This indicates that the efficacy of lamivudine was greatly influenced by the baseline factors of patients, which is a disadvantage of lamivudine treatment. However, the virological response of patients in the entecavir treatment group was in general not affected by liver function, HBV DNA load, HBeAg status, virus genotypes and other baseline factors, indicating the advantages of entecavir treatment.

A study was further performed on the relationship among CD3^+^, CD4^+^, CD8^+^, CD4^+^CD25^+^ and CD8^+^CD28^-^ frequency and virological response, as well as drug resistance, in both nucleoside therapy groups. The results indicated that the CD4^+^CD25^+^ levels in the 4-week virological response group and in the 2-year non-resistance of the lamivudine treatment group were higher than in the suboptimal response group and drug resistance group. This was in contrast with the opinion of many scholars that the immune suppression of CD4^+^CD25^+^ has a negative effect on the clearance of viruses (14–16). This analysis indicated that such results may be related to antiviral actions of partially activated effector T cells included in CD4^+^ CD25^+^ (17). Therefore, recent international and local studies have considered that specific T cells containing CTLA-4, GITR, OX-40 and FoxP3, as well as other surface markers, were more suitable for the features of Treg cells (18). Hence, some scholars considered that highly expressed FoxP3CD4^+^ CD25^+^T was a specific marker for Treg cells (4). No significant difference was found in CD4^+^CD25^+^T level between the virological response group and suboptimal response group in the entecavir treatment group. However, CD8^+^CD28^-^ level in the response group was significantly lower than in the suboptimal response group. In addition, CD8^+^CD28^-^ level in the non-resistance group with lamivudine treatment was significantly lower than that in the resistance group. This indicated that increased CD8^+^CD28^-^ levels reduced the clearance capacity of viruses and increased drug resistance risk. International scholars (19,20) have demonstrated that CD8^+^CD28^-^ could induce a specific T cell subgroup of tolerant APC, and result in no reactivity of helper T cell (Th) by triggering inhibitory signal circuits. This indicates that the immunosuppressive action of CD8^+^CD28^-^ is stronger and more extensive than that of CD4^+^CD25^+^. Accordingly, our study found that CD8^+^CD28^-^ percentage can more accurately reflect immune suppression on the clearance of HBV. Boni et al. (15) indicated that for patients who responded to nucleoside therapy effectively, CD4^+^CD8^+^ level remained lower than healthy people, and immune response in the patients with chronic HBV was weaker than in healthy people. These data indicate that partial actions of HBV-specific T lymphocytes were recovered, but without returning to normal levels.
